# Authors’ Response to Peer Reviews of “Rapidly Benchmarking Large Language Models for Diagnosing Comorbid Patients: Comparative Study Leveraging the LLM-as-a-Judge Method”

**DOI:** 10.2196/81235

**Published:** 2025-08-29

**Authors:** Peter Sarvari, Zaid Al-fagih

**Affiliations:** 1 Rhazes AI, First Floor, 85 Great Portland Street, London, W1W 7LT, United Kingdom

**Keywords:** LLM, GPT-4, Gemini, Claude, RAG, clinical medicine, diagnosis, diagnostic ability of LLMs, large language model, AI in medicine, AI in healthcare, retrieval-augmented generation, artificial intelligence

*This is the authors’ response to the peer-review report for “Rapidly Benchmarking Large Language Models for Diagnosing Comorbid Patients: Comparative Study Leveraging the LLM-as-a-Judge Method.”*

Many thanks for your thoughtful comments [1] on our submission [2]. We spent time to carefully rewrite the article so it addresses the editorial comments and those from the live review.

## Major Concerns and Suggested Improvements

### Title Revision

*The current title does not fully capture the scope of the study; hence, it needs to be reconsidered. It would be nice if abbreviations were avoided in the title. Therefore, we recommend the authors of the study change “LLM” to “Large Language Models” in the title.*

**Response:** Title has been revised.

### Abstract and Introduction Clarity

*The abstract and the introduction lack a clear statement of the study’s aim. It is therefore expedient to revise the abstract to include the objectives, methodology, key results, and conclusion. The Introduction should have a clear research aim.*

*Note: During the call, the authors shared that a revised version of the abstract had been generated and shared so it is possible that the latest version has addressed this concern. We invite the authors to share their updated version in the comment section of this review.*

**Response:** The abstract and introduction have been significantly rewritten since the live review.

### Physician Comparison With Large Language Models

*The study does not explore how diagnoses differ from physicians using only the data provided to the large language model (LLM). It would be advisable to include a comparative analysis to evaluate diagnosis accuracy and the prioritization of additional tests between physicians and LLMs.*

*Furthermore, the absence of actual data around patient history and other diagnostic parameters beyond what was reported in billing reports (reported as “ground truth” in the study) is a weakness. This can lead to an incomplete or partial diagnosis being labeled as the final diagnosis, leading to miscalculations about the accuracy of LLMs.*

**Response:** We added this as a limitation of the study.

### Model Selection Rationale and Evaluation Metrics

*The Methods section is limited in its description of the methodology used in the study. It would be helpful to include more information on the rationale for the model selection and describe differences between GPT-4 variants to help readers understand the comparative approach.*

*Furthermore, the choice of “hit rate” as the primary evaluation metric is unclear, and its limitations are not discussed in sufficient detail. It would be helpful if the choice of hit rate over other metrics (eg, precision or F1-score) as well as the limitations the hit rate may introduce were discussed more thoroughly.*

**Response:** We expanded the Methods section to include more details about the automated evaluation, retrieval-augmented generation (RAG), and the model versions used. The rationale for choosing hit rate is now described in detail in the methods.

### Methodology and RAG Integration Details

*The role of RAG in the diagnostic process, including how relevant information was retrieved and implemented to enhance the diagnostic process and performance needs to be elaborated further as it constitutes a novel part of the study. This issue was highlighted as one of the particular concerns, as without more details, many questions remain unanswered and that could compromise the credibility of the study.*

**Response:** We added more details on our RAG methodology, simplified and reran all experiments, and confirmed their statistical significance. The exact sections retrieved (10 out of 32 chunks) vary between the 1000 patients and experiment runs.

### Data Interpretation and Population-Specific Reference Ranges

*Reference ranges used for diagnoses are not adequately explained. The authors are encouraged to clarify if the reference ranges are population-specific or if they align with the dataset characteristics.*

*In general, reviewers suggest authors add more details about the nature of the data beyond referring to them as “test results” in the manuscript. For example, it would be helpful to know more about the meaning and interpretation of the homogeneity of the test results and the implications of it on the evaluation of the method.*

*Provide a statistical analysis to demonstrate that the differences in diagnostic hit rates for the LLMs are statistically significant in the range of 98.5-99.8.*

**Response:** We use the most recent American Board of Internal Medicine laboratory reference ranges as referenced. Please see [3].

### Discussion

*It would be helpful to discuss why GPT-4.0 and Claude 3.5 Sonnet performed better than others, potentially due to architectural differences or data training sources.*

*It would also be important to discuss why specific diagnoses (eg, diabetes) were among the best hits and most frequent misses.*

**Response:** Unfortunately, we do not have access to the exact architecture or training data of closed-source models provided by companies like OpenAI or Anthropic. The exact reasons the diagnostic models gave for hits and the assessor models gave for missed diagnoses are available from the GitHub repository directly. All results are saved as a CSV under the “data” folder. If the editors would like, we can give an example about how one would go about analyzing these in the appendix.

### Limitations of Study Design

*The limitations could be explicitly outlined in a separate section of the Discussion for transparency and clarity. For example, the authors may include a discussion around the fact that the sample size (1000 patients) may be too small to generalize the findings, potential issues related to relying on billing reports as ground truth, and considerations of hallucinations or failure scenarios of LLMs in real-world settings. In such a section, the authors may also explore ideas related to using larger and more diverse datasets in similar future research.*

**Response:** The Limitations section has been greatly expanded.

### Figures and Tables

*The figures and tables in the study lack clarity and, at times, key information (eg, patient demographics, disease types are missing). The authors are advised to add clarity to the data visualizations, label axes, and include interpretive analyses to all figures, but in particular for Tables 1 and 2, and Figure 2. They are also advised to discuss specific trends such as frequent misses for certain conditions.*

**Response:** Tables and figures have been changed significantly since this comment was made.

### Reproducibility

*The reproducibility of the study is hindered by the lack of clear documentation on LLM settings, dataset transformations, and code. It is suggested that the authors provide the full details of the LLM configurations, processing steps, and code availability.*

*For example, it would be helpful to know the rationale for limiting the LLM output tokens to 4096. How could this be relevant to the “human” diagnostic process? Were some predictions judged “more likely” than others?*

**Response:** Everything needed for reproducibility has been shared on the GitHub repository. If the editors would like, we can give an example about how one would go about running a new experiment in the appendix.

### Bias and Real-World Application

*Potential biases in LLM predictions and challenges in clinical adoption are not addressed in the study. It is advised that the authors add a section on potential biases and practical integration challenges. They need to include future work on improving model robustness and fairness.*

**Response:** Thank you—it was added to the Limitations section.

## Minor Concerns and Suggested Improvements

### Abbreviation Usage

*Key abbreviations (eg, LLM, RAG) were not defined at first use. The authors are encouraged to define all abbreviations when first indicated in the abstract and body of the study (eg, “electronic health records (EHRs)” when first mentioned, then “EHR” at later mentions).*

**Response:** Thank you—RAG and EHR have been defined.

### Language

*There are several typos and some grammatical errors, incomplete sentences, and contractions that reduce the readability of the study; hence, the authors are encouraged to consider thorough proofreading and editing to improve the reading experience and interpretation of the study. This is a minor concern that may be well addressed by the copyeditors of the journal that will publish the manuscript.*

**Response:** Thank you—we made further edits to make the manuscript more readable.

### Ethical Statement Clarity

*The ethical considerations for using MIMIC-IV data are not explicitly referenced. The authors should state that the dataset is deidentified and describe access restrictions for researchers. Some reviewers had concerns about the need for ethical approval given the use of patient data, but others reported that ethical approval may not be needed given the public nature of the data used.*

*Furthermore, it would be helpful to add a discussion around the potential risk of bias introduced by LLMs and its large implications on diagnosis and the field of medicine at large.*

**Response:** Added under Data Availability statement.

### False-Positive and False-Negative Rates

*The explanation of false-positive and false-negative rates in the study is inadequate; hence, the authors are invited to include specific examples and explanations of why certain diagnoses were misclassified.*

**Response:** A specific example was given in our previous study [4]. Could you please clarify which part of the explanation was inadequate?

### Conclusions

*The author should consider adding a section that examines potential biases in LLM predictions and the practical challenges of using these models in hospital settings. Furthermore, it would be helpful to further highlight practical takeaways or future directions, emphasizing actionable insights and specific areas for future research (eg, integrating multimodal data sources or fine-tuning models with diverse clinically annotated datasets).*

**Response:** While we did not add a separate section about biases, we direct the reader to a comprehensive review on this topic. Future directions about hospital implementation and improving the limitations have been addressed.

### Comparative Model Performance

*Performance differences between models in the study are not sufficiently elaborated on in the Discussion. The authors are invited to explore why certain models performed better, considering architectural differences and training data sources.*

*Reviewers also advised authors to consider human vetting for the evaluation to provide an additional layer of confidence to get the experts to reflect on the LLM answers and explanations.*

**Response:** Unfortunately, we do not have access to the exact architecture or training data of closed-source models provided by companies like OpenAI or Anthropic.

### Hyperbolic Language

*Words like “stunning” are overly subjective. The use of neutral language is advised in the manuscript, and the authors are invited to justify claims with supporting data.*

**Response:** Thank you—they have been removed.

### Dataset Limitations

*Rare diseases may not be adequately represented in the study. The authors should address how dataset limitations affect diagnostic performance and include rare disease cases in future studies.*

**Response:** The Limitations section has been greatly expanded.

### Citations to Methods and Tools

*Where possible, add citations to specific LLM and RAG tools used, such as technical references from Google, OpenAI, etc, to aid readers in finding more information on these tools.*

*Authors are advised to complete their statements instead of just including a citation. For example, “In this case, the further tests the LLM is instructed to suggest [2] are of crucial importance to understand exact disease pathology.”*

*Provide an explanation of the sentence “NEJM Case Challenges are notoriously hard” and provide a reference. Potentially, reconsider the use of the extreme adverb “notoriously”—perhaps “well known to be.”*

**Response:** Statements have been completed. We are happy to add references to all models mentioned in the article if the editors agree that this would enhance the quality of the paper. Most model documentations can be found simply by googling the model name and version, both of which we have provided. Extreme words have been removed according to our best judgment.

### Presentation of Methods

*For readability, reformat the list of LLMs used into a table with separate columns for name, version, and settings.*

**Response:** We are not sure that this would enhance the flow of the manuscript. If the editors agree, however, we are happy to make such a modification. The settings are largely synonymous across models; it is just the model names and versions that differ.

The authors would like to thank all peer reviewers for their thoughtful feedback and great contribution toward making this manuscript better.

## Abbreviations

EHR: electronic health record
LLM: large language model
RAG: retrieval-augmented generation
